# Mineral Metabolism Markers Are Associated with Myocardial Infarction and Hemorrhagic Stroke but Not Ischemic Stroke in Hemodialysis Patients: A Longitudinal Study

**DOI:** 10.1371/journal.pone.0114678

**Published:** 2014-12-10

**Authors:** Miho Tagawa, Takayuki Hamano, Hiroshi Nishi, Kenji Tsuchida, Norio Hanafusa, Atsushi Fukatsu, Kunitoshi Iseki, Yoshiharu Tsubakihara

**Affiliations:** 1 Department of Nephrology, Kyoto Katsura Hospital, Kyoto, Japan; 2 Committee of Renal Data Registry, Japanese Society for Dialysis Therapy, Tokyo, Japan; 3 Department of Nephrology, Kyoto University Graduate School of Medicine, Kyoto, Japan; Nanjing Medical University, China

## Abstract

**Background/Aims:**

The associations between phosphate, calcium, and intact parathyroid hormone (PTH) levels and composite cardiovascular end points have been studied. This study examined the associations of these markers with myocardial infarction (MI) and stroke separately.

**Methods:**

This is a longitudinal study on 65,849 hemodialysis patients from the Japan Renal Data Registry. Patients with prior events at baseline were excluded. Predictors were phosphate, albumin-corrected calcium, intact PTH, and calcium times phosphate product levels. Outcome was the first episode of MI or stroke during a 1-year observation period. Data were analyzed using multiple logistic regression analyses, adjusted for potential confounders.

**Results:**

There were 1,048, 651, and 2,089 events of incident MI, hemorrhagic, and ischemic stroke, respectively. Incident MI was associated with phosphate levels ≥6.5 mg/dL (odds ratio 1.49; confidence interval 1.23–1.80) compared with phosphate levels of 4.7–5.4 mg/dL and intact PTH levels>500 pg/mL (1.35; 1.03–1.79) compared with intact PTH levels of 151–300 pg/mL. Higher albumin-corrected calcium level was positively associated with MI (p = 0.04 by trend analysis). Hemorrhagic stroke was associated only with intact PTH levels>500 pg/mL (1.54; 1.10–2.17). Incident ischemic stroke had no association with phosphate, calcium, or intact PTH levels. The association of calcium times phosphate product with outcomes was essentially the same pattern as that of phosphate and outcomes.

**Conclusions:**

MI was associated with phosphate, calcium, and intact PTH levels, whereas hemorrhagic stroke was associated only with intact PTH. Ischemic stroke was not associated with any of them. The potential distinct beneficial effect on MI and stroke by managing bone and mineral disease should be investigated in future studies.

## Introduction

A number of studies have investigated the associations between serum phosphate (P), calcium (Ca), and intact parathyroid hormone (PTH) levels and mortality or a composite of cardiovascular events in dialysis patients [1]–[8]. These studies consistently showed that hyperphosphatemia, hypercalcemia, and high intact PTH levels were associated with increased mortality or an increased incidence of a composite of cardiovascular events. However, there has been a lack of data on the associations between P, Ca, and intact PTH levels and individual cardiovascular end points such as myocardial infarction (MI), hemorrhagic stroke, or ischemic stroke. There is a possibility that the effects of mineral metabolism on each individual cardiovascular end point could be distinct, but the distinct effect is masked when the composite outcome is used.

In this study, we hypothesized that the associations of serum P, Ca, and intact PTH levels with each separate cardiovascular outcome may vary. Histories of MI, hemorrhagic stroke, and ischemic stroke are collected separately in the Japanese Renal Data Registry (JRDR), which is the national registry of Japanese dialysis patients maintained by the Japanese Society for Dialysis Therapy (JSDT). We examined our hypothesis using the JRDR database from 2006 to 2007.

## Materials and Methods

### Study Design

This is a longitudinal study based on the JRDR database from 2006 to 2007. Details on the JRDR have been published previously [9]–[11]. Briefly, it is a database of all incident and prevalent dialysis patients in Japan. At the end of every year, the staff of each dialysis unit voluntarily participates in data collection. The response rates were 94.0% and 94.8% in 2006 and 2007, respectively.

Notably, cinacalcet was approved in Japan in January 2008 and was therefore not available during the study period.

### Subjects

The inclusion criteria were as follows: subjects (1) on hemodialysis for more than a year at the end of 2006, (2) aged 20–99 years, and (3) whose survival status was available at the end of 2007. The exclusion criteria were as follows: subjects (1) with missing data, (2) on peritoneal dialysis, (3) who underwent renal transplantation in 2007, or (4) with implausible data or apparent errors (e.g., intradialytic weight loss [predialysis weight − postdialysis weight]>10 kg, intradialytic weight gain [possible fluid administration during dialysis]>5 kg, or a predialysis diastolic blood pressure greater than the predialysis systolic blood pressure). As the JSDT collects data on the histories of MI and stroke at the end of each year, whether subjects had events during 2007 could not be determined for those with histories of MI or stroke at the end of 2006. Thus, subjects with histories of MI, hemorrhagic stroke, and ischemic stroke at baseline were excluded from the analyses when the outcome variables were incident MI, hemorrhagic stroke, and ischemic stroke, respectively.

### Predictors

Predictor variables were predialysis serum P, albumin-corrected Ca (cCa), intact PTH, and calcium times phosphate product levels at the end of 2006. Of note, it is the standard of care in Japan to evaluate these parameters at the first dialysis session of the week, and this is specified in the JRDR. The cCa was calculated as follows: cCa  =  serum calcium + [4 − serum albumin (g/dL)] if serum albumin is less than 4 g/dL [12]. Calcium times phosphate product was calculated using cCa levels [1]. Sensitivity analysis was performed for calcium times phosphate product calculated with uncorrected Ca [3].

### Outcomes of Interest

Outcome variables were the first episodes of MI, hemorrhagic stroke, and ischemic stroke during the 1-year observation period in 2007. Subjects were considered to have incident events if they had no histories of these diseases in the 2006 database and did have histories of these diseases in the 2007 database.

### Statistical Analyses

To calculate the incident rate of each cardiovascular disease outcome, incident MI, hemorrhagic stroke, and ischemic stroke were considered to have occurred 6 months into the follow-up period because the dates of the events were not available.

Data were evaluated using a multiple logistic regression analyses adjusted for the following variables: age; sex; dialysis vintage; causes of end-stage renal disease (diabetic nephropathy, hypertensive nephrosclerosis, chronic glomerulonephritis, and others); categories of dialysis frequency (1–2, 3, and 4–6 sessions/week) and dialysis duration (<4, 4, and>4 hours/session); quintiles of predialysis serum albumin level, total cholesterol level, body mass index, *Kt*/*V*, protein catabolic rate, ultrafiltration rate [(predialysis body weight − postdialysis body weight)/hours of dialysis/postdialysis body weight], and hemoglobin level; categories of vascular access (arteriovenous fistula, arteriovenous graft, central venous catheter, and others); 5 categories of predialysis systolic blood pressure (≤120, 121–140, 141–160, 161–180, and>180 mm Hg) and predialysis diastolic blood pressure (≤60, 61–80, 81–90, 91–110, and>110 mm Hg); and antihypertensive use (angiotensin-converting enzyme inhibitors, angiotensin receptor blockers, calcium channel blockers, and others). In addition, when incident MI was an outcome variable, histories of hemorrhagic and ischemic strokes at the end of 2006 were used as covariates. Similar adjustments were performed when incident hemorrhagic and ischemic strokes were outcome variables. When calcium times phosphate product was a predictor variable, cCa and P were excluded from the analyses as these variables were strongly correlated with calcium times phosphate product. Logistic regression analyses were performed instead of survival analyses because the data indicated that the subjects had histories of each disease at the end of the year, but not when the events occurred during the year. Predialysis blood pressure and antihypertensive use were imputed from the 2005 database (last observation carried forward) as these variables were not available in the 2006 database. Trend analyses were also performed. Restricted cubic spline analyses were performed for incident MI, which showed significant associations with P, cCa, and intact PTH levels, with adjustment for the same covariates, because the cutoff values for categories of P, cCa, and intact PTH levels were determined from current guidelines [13] and the ability to detect significant differences between categories could be affected by the selection of cutoff values.

Statistical analyses were performed using SPSS version 19.0 (SPSS Inc, Chicago, IL), with the exception of cubic spline analyses, which were performed using Stata version 11.1 (StataCorp, College Station, TX).

### Ethics Statement

The study protocol was approved by the Medicine Ethics Committee of the Japanese Society for Dialysis Therapy. The complete de-identification has secured the privacy of the human subjects in our database, its secondary or unofficial use (i.e. any distribution to a third party, unauthorized replication or manipulation of database, and deviation from the proposal accepted by the Committee of Renal Data Registry) is strictly prohibited by the provision of agreements between the principle investigators and the Japanese Society for Dialysis Therapy, by which all rights regarding the database are reserved.

## Results

**Figure 1:**
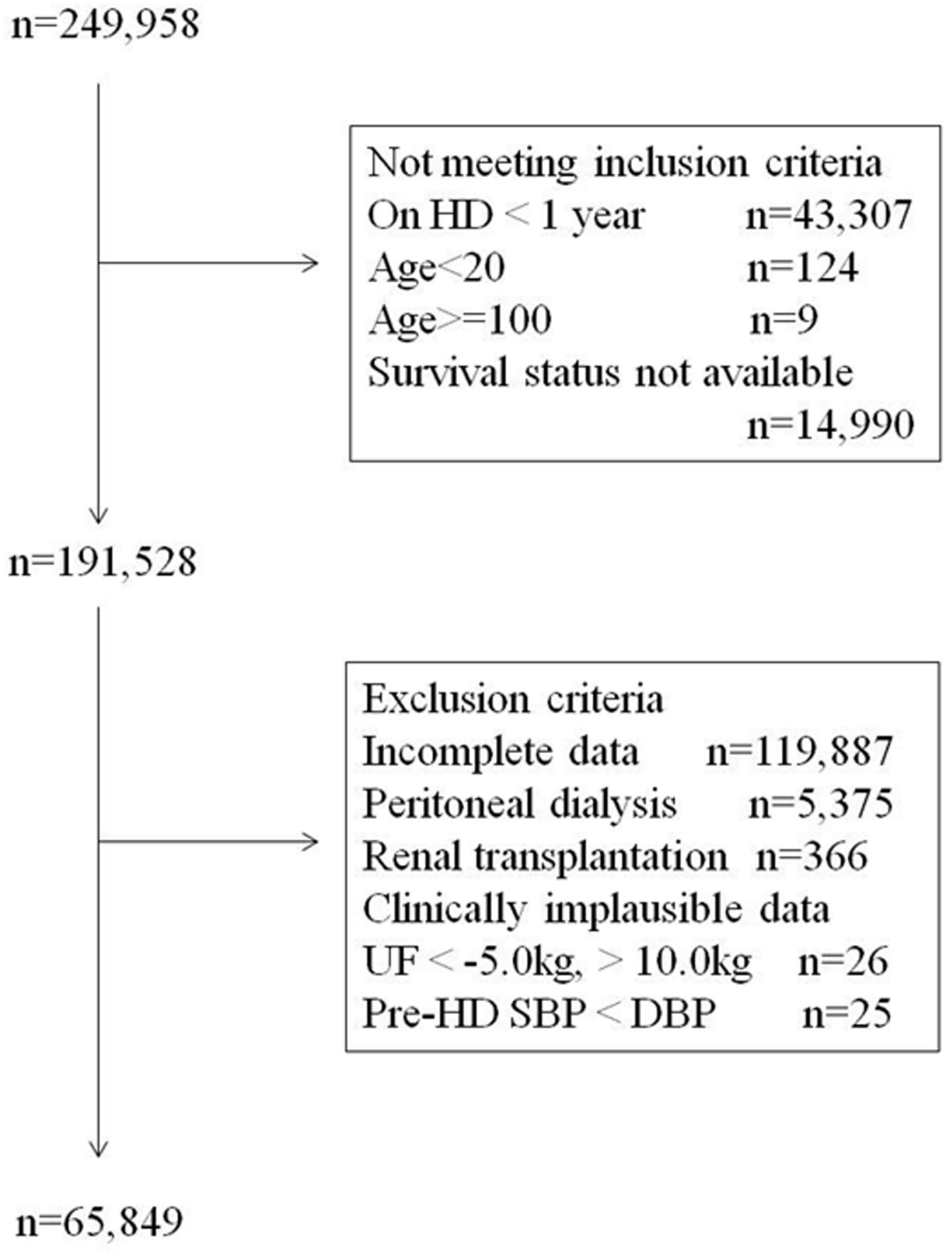
Flow chart of patients. HD: hemodialysis, UF: ultrafiltration, SBP: systolic blood pressure, DBP: diastolic blood pressure.

**Figure 2:**
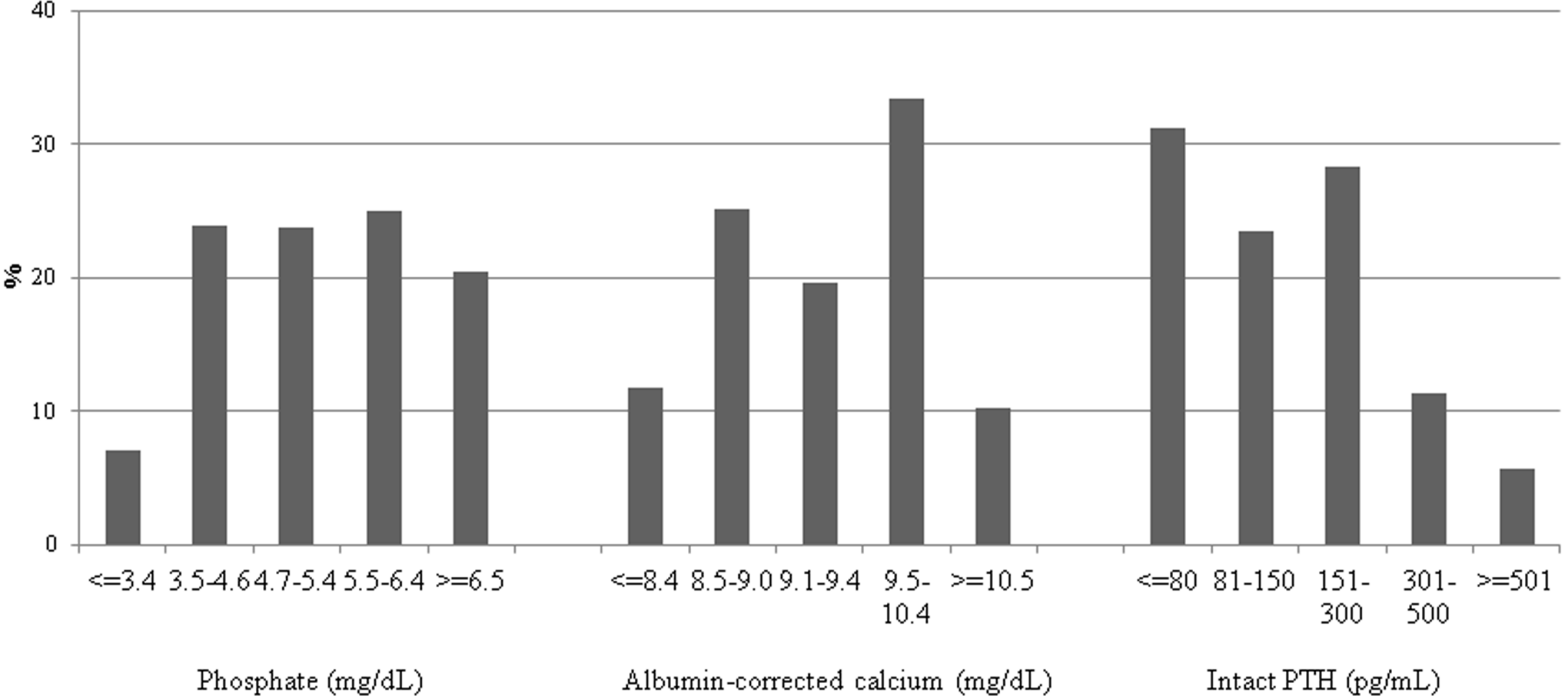
Distribution of pre-dialysis serum phosphate, albumin-corrected calcium, and intact PTH levels. PTH: parathyroid hormone.

At the end of 2006, data were collected on 249,958 patients treated in 3,985 dialysis facilities in Japan. Among them, 191,528 patients met the inclusion criteria. After the exclusion of patients with incomplete data, those on peritoneal dialysis, those who underwent renal transplantation during 2007, and those with clinically implausible data, data from 65,849 patients were analyzed (Fig. 1). The characteristics of the subjects included in the analysis are shown in Table 1. The distributions of predialysis P, cCa, and intact PTH levels are shown in Fig. 2. In the total patient sample, 23.7% had a P level of 4.7–5.4 mg/dL, 19.6% had a cCa level of 9.1–9.4 mg/dL, and 28.3% had an intact PTH level of 151–300 pg/mL, which were used as reference categories. Compared with data from the United States and Europe, the proportion of patients in our sample with a PTH level ≤80 pg/mL was much higher and the proportion of patients with a PTH level ≥501 pg/mL was much lower [3], [4], [7].

**Table 1:** Demographics.

Age	63.5 (12.3)
Male sex	39,627 (60.2)
Dialysis vintage (years)	7.0 (4.0–12.1)
Causes of end-stage renal diseases	
Hypertensive nephrosclerosis	4,228 (6.4)
Diabetic nephropathy	19,048 (28.9)
Chronic glomerulonephritis	30,989 (47.1)
Others	11,584 (17.6)
Frequency of dialysis	
1–2/week	1,938 (2.9)
3/week	63,685 (96.8)
4–7/week	226 (0.3)
Duration of each dialysis treatment	
<4 hr	12,431 (18.9)
4 hr	43,820 (66.5)
>4 hr	9,598 (14.6)
Albumin (g/dL)	3.8 (0.4)
Total cholesterol (mg/dL)	154 (36)
Body mass index	21.2 (3.9)
Kt/V	1.41 (0.28)
Protein catabolic rate	0.92 (0.18)
Ultrafiltration rate (mL/hr/kg)	11.6 (4.4)
Vascular access	
Arteriovenous fistula	59,978 (91.1)
Arteriovenous graft	4,474 (6.8)
Central venous catheter	195 (0.3)
Others*	1,202 (1.8)
Pre-dialysis systolic blood pressure	
< = 120 mmHg	5,355 (8.1)
121–140 mmHg	14,406 (21.9)
141–160 mmHg	22,488 (34.1)
161–180 mmHg	16,105 (24.5)
> = 181 mmHg	7,495 (11.4)
Pre-dialysis diastolic blood pressure	
< = 60 mmHg	6,062 (9.2)
61–80 mmHg	32,815 (49.8)
81–90 mmHg	15,532 (23.6)
91–110 mmHg	10,516 (16.0)
> = 111 mmHg	924 (1.4)
Antihypertensives	
Calcium channel blockers	33,644 (51.1)
Angiotensin converting enzyme inhibitors	7,603 (11.5)
Angiotensin receptor blockers	23,583 (35.8)
Others	18,632 (28.3)

*This includes subcutaneously fixed superficial arteries and direct puncture of arteries.

Data shown as mean (SD) or median (interquartile range) for continuous variables and number (%) for categorical variables.

The prevalence and incidence of mortality and cardiovascular morbidity are shown in Table 2. The incidence of ischemic stroke was highest among the 3 events studied, followed by MI and hemorrhagic stroke.

**Table 2:** Prevalence and incidence of mortality and cardiovascular morbidity.

	Prevalence of events at the end of 2006Number (%)	Incident events during 2007 in subjects without prior events	Events/100 person-years*
All-cause mortality		387	0.6
Myocardial infarction	3,602 (5.5)	1,048	1.6
Hemorrhagic stroke	2,323 (3.5)	651	1.0
Ischemic stroke	7,485 (11.4)	2,089	3.6
Composite of myocardial infarction and stroke	11,854 (18.0)	3,014	5.7

*Incident myocardial infarction, hemorrhagic stroke, ischemic stroke were considered to have occurred at 6 months of the follow-up period.

**Figure 3:**
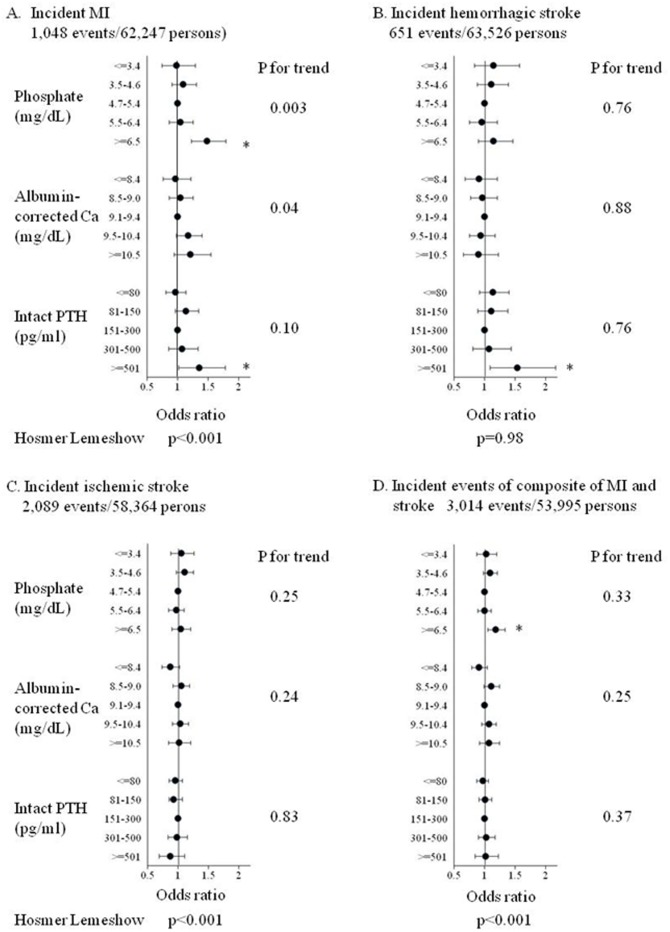
Association between categories of pre-dialysis serum phosphate, albumin-corrected calcium, intact PTH levels and incident MI (A), hemorrhagic stroke (B), ischemic stroke (C), and composite of MI and stroke (D). MI: myocardial infarction, Ca: calcium, PTH: parathyroid hormone.

**Figure 4:**
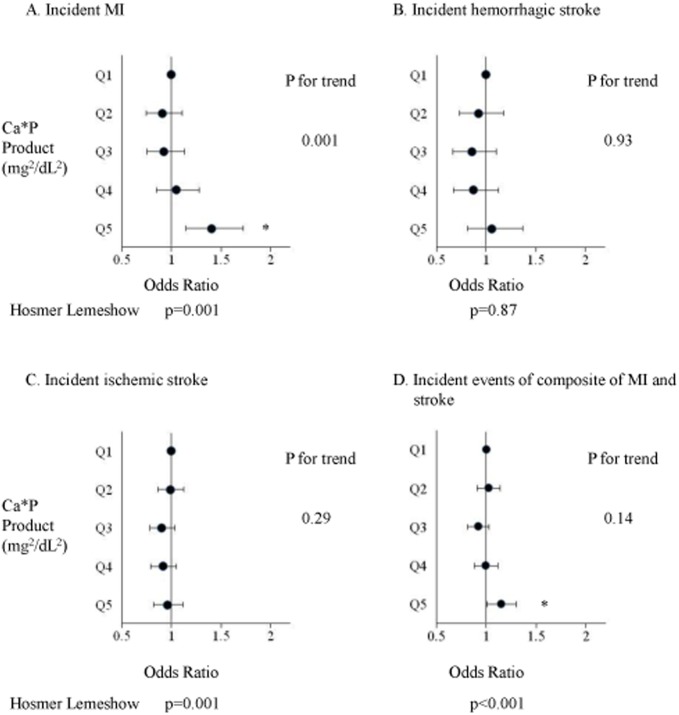
Association between quintiles of calcium times phosphate product and incident MI (A), hemorrhagic stroke (B), ischemic stroke (C), and composite of MI and stroke (D). MI; myocardial infarction, Ca*P product: calcium times phosphate product. Calcium times phosphate product levels in each quintiles were as follows: Q1: <37.4, Q2: 37.4–44.6,Q3: 44.7–51.5, Q4: 51.6–60.3, Q5:>60.3 mg^2^/dL^2^.

The associations between 5 categories of P, cCa, and intact PTH levels and incident MI, hemorrhagic stroke, and ischemic stroke after adjustment for covariates are shown in Fig. 3A–C. MI was significantly associated with hyperphosphatemia, higher cCa levels, and high intact PTH levels. The odds ratio (OR; 95% confidence interval [CI]) for incident MI was 1.49 (1.23–1.80) for those with a P level ≥6.5 mg/dL compared with a P level of 4.7–5.4 mg/dL. The association between P categories and incident MI was also statistically significant by trend analysis (p = 0.003). An intact PTH level>500 pg/mL was significantly associated with incident MI compared with an intact PTH level of 151–300 pg/mL (OR, 1.35; 95% CI, 1.03–1.79). Although none of the cCa categories were significantly associated with incident MI, the p-value by trend analysis was 0.04, indicating a higher incidence of MI in patients with higher calcium levels. Hemorrhagic stroke was significantly associated only with a high intact PTH level. An intact PTH level>500 pg/mL was significantly associated with incident hemorrhagic stroke compared with an intact PTH level of 151–300 pg/mL (OR, 1.54; 95% CI, 1.10–2.17). P and cCa categories were not associated with incident hemorrhagic stroke. Ischemic stroke was not associated with P, cCa, and intact PTH categories. The associations between the 5 categories of P, cCa, and intact PTH levels, and incident events of the composite of MI and stroke after adjustment for covariates are shown in Fig. 3D. The composite of MI and stroke was associated only with hyperphosphatemia. A P level ≥6.5 mg/dL was significantly associated with incident events of the composite of MI and stroke compared with a P level of 4.7–5.4 mg/dL (OR, 1.19; 95% CI, 1.06–1.34). Categories of cCa and intact PTH levels were not associated with the composite of MI and stroke. The pattern of association between calcium times phosphate products and outcomes was essentially the same as that of P and outcomes (Fig. 4). Sensitivity analysis using calcium times phosphate product calculated with uncorrected Ca did not significantly change the results (data not shown).

**Figure 5:**
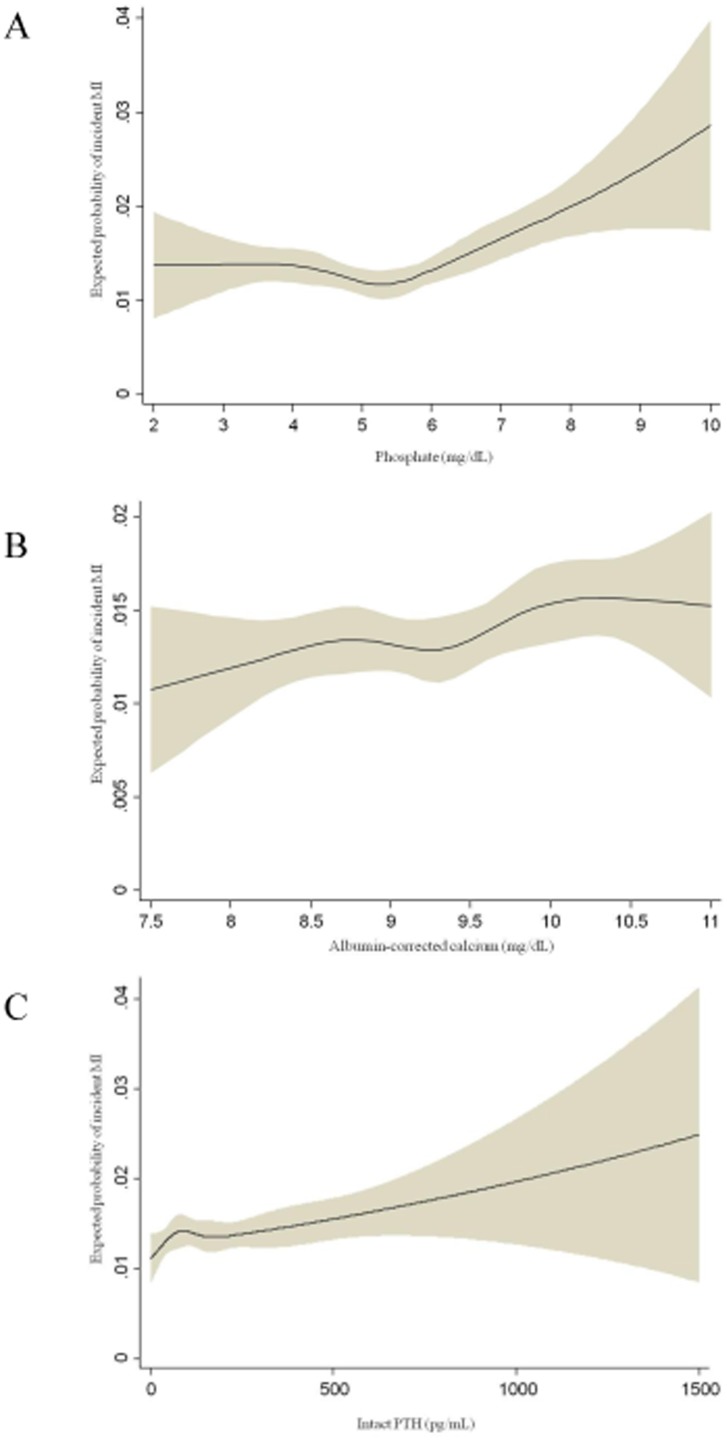
Expected probability of MI according to pre-dialysis serum phosphate (A), albumin-corrected calcium (B), and intact PTH (C) levels by restricted cubic spline analyses. MI: myocardial infarction, PTH; parathyroid hormone.

Restricted cubic spline analyses were performed for incident MI, which had the greatest associations with P, cCa, and intact PTH levels (Fig. 5). The expected probability of incident MI was lowest around a P level of 4.5–6 mg/dL. A monotonous increase in the expected probability of incident MI was associated with increases in cCa and intact PTH levels.

## Discussion

In this study, the associations between P, cCa, and intact PTH levels, and various cardiovascular diseases (MI, hemorrhagic stroke, and ischemic stroke) were examined separately. Our major findings were that among the 3 end points of MI, hemorrhagic stroke, and ischemic stroke, P, cCa, and intact PTH levels had the greatest associations with incident MI. Hyperphosphatemia, higher calcium, and high intact PTH levels were associated with incident MI. The risk of incident MI associated with an intact PTH level>500 pg/mL was similar to that associated with a P level ≥6.5 mg/dL (1.35, 1.03–1.79 and 1.49, 1.23–1.80, respectively). High intact PTH levels, but not P or cCa levels, were associated with incident hemorrhagic stroke. P, cCa, and intact PTH levels were not associated with incident ischemic stroke. Hyperphosphatemia was associated with incident events of the composite of MI and stroke. However, the composite of MI and stroke was not associated with high intact PTH, even though high intact PTH levels were significantly associated with incident MI and hemorrhagic stroke when these end points were analyzed separately.

Regarding the first finding (associations of mineral metabolism markers with MI and hemorrhagic stroke but not with ischemic stroke), the absence of an association with ischemic stroke was not caused by a lack of statistical power, as the incidence of ischemic stroke was higher than that of MI or hemorrhagic stroke. Despite the low incidence of hemorrhagic stroke in our sample and hence low statistical power, a significant association with intact PTH levels>500 pg/mL was detected. One previous study [14] showed that a P level>6.5 mg/dL compared with 2.4–6.5 mg/dL and calcium times phosphate product (per 10 mg^2^/dL^2^) was associated with a significant increase in the relative risk of death due to coronary artery disease but not death due to cerebrovascular accident. These results are consistent with our findings of an association of hyperphosphatemia with incident MI only, but not with stroke. This prior study did not examine the associations of calcium and intact PTH with coronary artery disease or cerebrovascular accident.

Why MI is associated with mineral metabolism markers but ischemic stroke is not? Although vascular calcification has been widely accepted to link the derangement of mineral metabolism and cardiovascular morbidity in hemodialysis patients through arterial stiffness and left ventricular hypertrophy [15], medial calcification in these patients does not cause instability or rupture of coronary plaque, which is a pathophysiological mechanism of MI [16]. It has been reported that phosphate causes endothelial dysfunction. In vitro, endothelial cells cultured at phosphate concentrations seen in chronic kidney disease (2.5 mM) display impaired nitric oxide synthesis [17] and increased reactive oxygen species generation and apoptosis [18]. In healthy human subjects, oral phosphate supplementation was associated with a transient reduction in endothelium-dependent brachial artery dilatation [17]. Phosphate and PTH are also reportedly associated with inflammatory markers such as C-reactive protein and interleukin-6 [19]. The PTH receptor is expressed on endothelial cells, vascular smooth muscle cells, and cardiomyocytes. In vitro, PTH upregulates endothelial cell expression of the proinflammatory and atherosclerotic mediators interleukin-6 and receptor of advanced glycation end products [20]. Since endothelial dysfunction is a precursor of atherosclerosis and is associated with coronary events [21], [22] and because inflammation plays an important role in the pathogenesis of MI [16], these findings may explain the links between P and PTH levels, and MI. In our study, the data could not be adjusted for C-reactive protein levels, as these were not available. The mechanisms for the association between higher calcium and MI need to be elucidated. On the other hand, it has been reported that the incidence of ischemic stroke is high during and shortly after hemodialysis. Onoyama et al. [23] reported that 64% of ischemic strokes in maintenance hemodialysis patients occurred during or 6 hours after hemodialysis. Toyoda et al. [24] also reported that in 34% of cases, the onset of ischemic stroke took place during or 30 min after hemodialysis, which was much higher than the incidence found in a similar examination of hemorrhagic stroke. In addition, in the Trial to Reduce Cardiovascular Events with Aranesp Therapy study, the use of darbepoetin to maintain hemoglobin levels at approximately 13 g/dL in diabetic patients with chronic kidney disease was associated with a significant increase in stroke but not MI or myocardial ischemia [25]. These previous studies suggest that hemodynamic change and hemoconcentration due to ultrafiltration during hemodialysis may be important risk factors for ischemic stroke. On the other hand, plaque rupture is the pathophysiological mechanism of MI rather than hemodynamic change [16]. Considering the results of these previous studies and differences in pathophysiology between coronary artery disease and cerebrovascular disease, it is possible that P, cCa, and intact PTH levels impact these 2 types of disease differently.

To our knowledge, the association of high intact PTH levels with hemorrhagic stroke has not been reported in previous studies. The reasons for this association were not clear. Previous studies reported that higher blood pressure, African American descent, sex, and lower *Kt*/*V* were associated with hemorrhagic stroke [26], [27] in hemodialysis patients. In our study, only Japanese patients were included and the data were adjusted for systolic and diastolic blood pressures, sex, and *Kt*/*V*. Anticoagulation with warfarin for atrial fibrillation may also be a predictor of hemorrhagic stroke in hemodialysis patients [28], [29]. However, intact PTH levels were not found to be associated with the prevalence of atrial fibrillation [28].

Regarding the second finding (association of composite of MI and stroke only with P, but not with Ca or intact PTH despite the significant association of MI with P, Ca and intact PTH, and hemorrhagic stroke with intact PTH), this is reasonable given that ischemic stroke was the event with the highest incidence in our sample; hence, the associations of the composite of MI and stroke were driven by the associations of ischemic stroke. This result is different from previous studies, consistently showing that hyperphosphatemia, hypercalcemia, and high intact PTH were associated with composite cardiovascular events [3], cardiovascular mortality [4], [5], or all-cause mortality [1]–[8]. The possible reasons for the inconsistent results are as follows: In the United States and Europe, the prevalence of coronary artery disease has been much higher than that in Japan [30]. Thus, associations of these markers with coronary artery disease may be reflected in cardiovascular composite outcomes in the United States and in Europe to a greater extent than in Japan. However, these associations have also been reported in 2 studies conducted in Japanese dialysis patients [5], [6]. In our study, only MI and stroke were included as outcomes, but in these previous studies, congestive heart failure, arrhythmia, and sudden cardiac death were also included [1]–[8]. Because congestive heart failure and sudden cardiac death are major causes of mortality in hemodialysis patients, the fact that these events were not included as outcomes may have led to differences in study results.

The strength of our study is that it is the largest study of the association between P, cCa, and intact PTH levels, and each individual cardiovascular disease. The JRDR database includes most dialysis patients in Japan. In addition, cinacalcet was not yet available in the market in Japan during the study period and therefore did not influence our results.

There are limitations to our study. As this study was observational, unknown confounders might have existed. In the database, the histories of MI, hemorrhagic stroke, and ischemic stroke were collected, but the direct incidences of these events were not. Thus, we could not identify recurrent events during the observational period in patients with prior events, who were excluded from the analysis. Because patients with prior cardiovascular events are at higher risk of developing subsequent events, the incident rates of each cardiovascular disease were grossly underestimated. There was no data on exactly when the events occurred during 1 year observation period. In addition, the incidence of congestive heart failure and sudden cardiac death, which are major cardiovascular comorbidities in hemodialysis patients, was not available in the database. The database was collected through the voluntary cooperation of the staff of each dialysis unit and large number of patients were excluded due to incomplete data, which might have introduced selection bias. There were no standardized criteria for the definitions of MI, hemorrhagic stroke, or ischemic stroke, so the quality of the data was not assured. Thus, there may be a misclassification bias, although the misclassification should be a random process irrespective of serum levels of P, cCa, or intact PTH. Furthermore, P, cCa, and intact PTH levels were surveyed only at one point at the end of 2006. These measurements may not necessarily represent the trend of these variables over the course of a whole year. The assays for iPTH were not standardized though approximately 80% of medical facilities in Japan used ELECSYS assay for intact PTH measurements (Roche Diagnostics, Mannheim, Germany) (personal communication with Dr. Hitoshi Kato, who was involved in developing clinical guideline for the management of mineral and bone disease in chronic kidney disease for Japanese Society for Dialysis Therapy). Also, the assumption that blood pressure and antihypertensives were the same as at the end of 2005 and 2006 was a limitation.

In conclusion, this study showed that hyperphosphatemia, higher calcium, and high intact PTH levels were significantly associated with incident MI and that high intact PTH levels were significantly associated with incident hemorrhagic stroke in hemodialysis patients. Only hyperphosphatemia was significantly associated with the composite of MI and stroke. These results suggest that when the associations of P, cCa, and intact PTH with cardiovascular events are investigated, each disease must be studied separately rather than as a composite outcome. These results are also hypothesis-generating and potential distinct benefit of treating mineral and bone disease on the risk of MI and stroke should be studied in future studies. Further studies are necessary to confirm these findings and elucidate the associations between mineral and bone diseases, and congestive heart failure, arrhythmia, and sudden cardiac death.
